# Enzymatically modified isoquercitrin supplementation intensifies plantaris muscle fiber hypertrophy in functionally overloaded mice

**DOI:** 10.1186/s12970-017-0190-y

**Published:** 2017-09-02

**Authors:** Akiko Kohara, Masanao Machida, Yuko Setoguchi, Ryouichi Ito, Masanori Sugitani, Hiroko Maruki-Uchida, Hiroyuki Inagaki, Tatsuhiko Ito, Naomi Omi, Tohru Takemasa

**Affiliations:** 1 Healthcare division, Morinaga & Co., Ltd., Yokohama, Kanagawa 230–8504 Japan; 2 Health and Sport Sciences, Graduate School of Comprehensive Human Sciences, University of Tsukuba, Tsukuba, Japan; 3 Organization of General Education, Saga University, Saga, Japan

**Keywords:** Enzymatically modified isoquercitrin, Rutin, Quercetin, Muscle, Hypertrophy, Functional overload, Whey protein

## Abstract

**Background:**

Enzymatically modified isoquercitrin (EMIQ) is produced from rutin using enzymatic hydrolysis followed by treatment with glycosyltransferase in the presence of dextrin to add glucose residues. EMIQ is absorbed in the same way as quercetin, a powerful antioxidant reported to prevent disused muscle atrophy by targeting mitochondria and to have ergogenic effects. The present study investigated the effect of EMIQ on skeletal muscle hypertrophy induced by functional overload.

**Methods:**

In Study 1, 6-week-old ICR male mice were divided into 4 groups: sham-operated control, sham-operated EMIQ, overload-operated control, and overload-operated EMIQ groups. In Study 2, mice were divided into 3 groups: overload-operated whey control, overload-operated whey/EMIQ (low dose), and overload-operated whey/EMIQ (high dose) groups. The functional overload of the plantaris muscle was induced by ablation of the synergist (gastrocnemius and soleus) muscles. EMIQ and whey protein were administered with food. Three weeks after the operation, the cross-sectional area and minimal fiber diameter of the plantaris muscle fibers were measured.

**Results:**

In Study 1, functional overload increased the cross-sectional area and minimal fiber diameter of the plantaris muscle. EMIQ supplementation significantly increased the cross-sectional area and minimal fiber diameter of the plantaris muscle in both the sham-operated and overload-operated groups. In Study 2, EMIQ supplementation combined with whey protein administration significantly increased the cross-sectional area and minimal fiber diameter of the plantaris muscle.

**Conclusion:**

EMIQ, even when administered as an addition to whey protein supplementation, significantly intensified the fiber hypertrophy of the plantaris muscle in functionally overloaded mice. EMIQ supplementation also induced fiber hypertrophy of the plantaris in sham-operated mice.

## Background

Rutin is a flavonoid glycoside that is ubiquitously present in a variety of fruits and vegetables, such as onions and buckwheat. Rutin is converted into quercetin and its metabolites before absorption [1]. Rutin and quercetin are known as vitamin P and play various roles in cardiovascular and metabolic disease [2]. As for the role of rutin in muscle, Mukai et al. reported that quercetin injection suppressed muscle atrophy by attenuating the induction of ubiquitin ligases [3]. They also found that quercetin intake prevented muscle atrophy by targeting mitochondrial function [4]. According to Seo et al., rutin intake increased the size of mitochondria and mitochondrial DNA content, as well as stimulated mitochondrial biogenesis [5]. Therefore, rutin and quercetin have a beneficial effect in muscle atrophy. It has also been reported that quercetin supplementation has an ergogenic effect [6]. However, the effects of rutin and its metabolites in muscle hypertrophy have remained unknown.

**Fig. 1:**
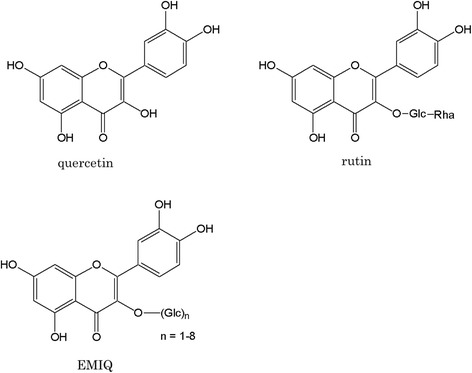
Chemical structures

Rutin and quercetin are poorly absorbed when administered orally, which diminishes their positive health effects [1]. Therefore, several trials have been conducted to increase the bioavailability of these compounds. Since quercetin is nearly insoluble in water (<0.1 g/100 mL at 21 °C), enzymatic glucosyl conjugation has been performed to enhance its water solubility [7, 8]. Enzymatically modified isoquercitrin (EMIQ) (Fig. 1) is one of water soluble glucoside derivatives of quercetin. EMIQ is produced from rutin via enzymatic hydrolysis, which removes the rhamnosyl group, followed by treatment of the product with glycosyltransferase in the presence of dextrin to add glucose residues. The bioavailability of EMIQ, which is absorbed as quercetin and metabolized like rutin, is about 17-fold greater than that of quercetin [8]. It is therefore reasonable to expect considerably greater health benefits with EMIQ. In Japan, EMIQ is approved as a food additive [9], and the U.S. FDA concluded that EMIQ is generally regarded as safe (GRAS) for use as an antioxidant. The level of EMIQ in a food product is recommended to be no greater than 150 mg/kg, with the exception of chewing gum, in which the antioxidant may be present up to 1500 mg/kg [10].

Skeletal muscle mass is maintained by a balance between synthesis and breakdown, with disproportionally increased protein synthesis leading to muscle fiber hypertrophy. Resistance training is known to induce muscle fiber hypertrophy, which is important to enhance exercise capacity and locomotive power in athletes and the elderly [11]. Protein and especially essential amino acids supplementation augments the muscle hypertrophy during resistance training [12, 13]. During resistance training, many athletes consume whey protein, which is known to augment muscle hypertrophy.

In this study, we used a mouse model of functional overload created by synergist ablation surgery. Surgical removal of the gastrocnemius and soleus muscles resulted in functional overload of the remaining plantaris muscle, leading to myofiber hypertrophy mimicking the effect of resistance training [14]. The purpose of this study was to evaluate the effect of EMIQ supplementation on muscle hypertrophy in these functionally overloaded mice. The effect of EMIQ supplementation simultaneously with whey protein was also evaluated.

## Methods

### Animals and experimental design

Male ICR mice (6 weeks old, *n* = 59) were obtained from Clea Japan Inc. Animals were maintained under standard conditions (temperature: 23 ± 2 °C, humidity: 50 ± 10%, 12:12-h light-dark cycle; lights on at 7:00 a.m.) with ad libitum access to food (Study 1: CE2, Clea Japan Inc.; Study 2: CE2 and AIN-93G, Oriental Yeast Co.) and water. After a 14-day acclimatization to the laboratory conditions, ablation of the synergistic gastrocnemius and soleus muscles was performed, and mice were maintained on standard bedding for one week. Thereafter, mice were individually housed on wire floor. They lived as they did in the cage previously. The body weight and food intake in each group were measured thrice a week. All animal experiments were carried out in a humane manner after receiving approval from the Institutional Animal Experiment Committee of the University of Tsukuba (identification number: 09–058) and in accordance with the Regulations for Animal Experimentation of the University and Fundamental Guidelines for Proper Conduct of Animal Experiments and Related Activities in Academic Research Institutions under the jurisdiction of Ministry of Education, Culture, Sports, Science and Technology of Japan.

#### Surgical procedure

In order to initiate overload-induced hypertrophy of the plantaris muscle, ablation of the synergistic gastrocnemius and soleus muscles was performed as described previously [15, 16]. Briefly, under pentobarbital sodium anesthesia (0.5%; 10 μL/g body weight), a skin incision was made from the popliteal to the Achilles tendon. The soleus muscle was completely removed except for a small portion at the proximal end, where it attaches to the plantaris. Both the lateral and medial gastrocnemius muscles were also completely removed. Care was taken to avoid trauma to the plantaris. In control groups, a sham operation was performed by making the skin incision only. The animals were sacrificed 3 weeks after the surgery, and the plantaris muscles of both hind limbs were excised and measured for weight. The samples were embedded in tissue-freezing medium (OCT compound, Sakura Finetek Japan), flash-frozen in liquid nitrogen, and stored at −80°C until use.

### EMIQ supplementation

EMIQ was obtained from Sanei-gen FFI Inc. The dosage of EMIQ supplementation was determined according to the previous study [17]. EMIQ was administered with diet at an average dose of approximately 4.0 mg/kg of body weight in study 1, and approximately 4.0 and 40 mg/kg of body weight in +EMIQ (L) and +EMIQ (H) groups, respectively, in study 2.

Study 1. The mice were randomly divided into four groups: sham-operated control (“Sham,” *n* = 9), sham-operated EMIQ (“Sham + EMIQ,” *n* = 9), overload-operated control (“Overload,” *n* = 10), and overload-operated EMIQ (“Overload + EMIQ,” *n* = 9). The control mice were kept on a normal diet (CE-2, Clea Japan Inc.), whereas mice in the EMIQ groups received the same diet and EMIQ (0.003%, Clea Japan Inc.) for 3 weeks (Table 1).

**Table 1:** Compositions of diets in Study 1

Ingredients(%)	CE-2	CE-2 + EMIQ
Carbohydrate	50.500	50.490
Protein	25.000	24. 995
Fat	4.700	4.699
Mineral mixture	6.800	6.799
Fiber	4.000	3.999
Water	9.000	8. 998
EMIQ	-	0.003
Total	100	100
Energy (kcal/100 g)	344	344

Study 2. The mice were randomly divided into three groups: overload-operated control mice that received whey protein supplementation (“Overload + W,” *n* = 7), overload-operated mice that received whey protein and a low dose of EMIQ (“+EMIQ (L),” *n* = 7), and overload-operated mice that received whey protein and a high dose of EMIQ (“+EMIQ (H),” *n* = 8). The mice in the whey control group received a diet in which milk casein in AIN-93G was replaced with whey protein (Clea Japan Inc.), whereas the mice in the whey EMIQ groups were fed the same whey diet and EMIQ (low dose: 0.003%; high dose: 0.03%) for 3 weeks (Table 2).

**Table 2:** Compositions of diets in Study 2

Ingredients(%)	Whey	+EMIQ(L)	+EMIQ(H)
Cornstarch	39.7486	39.7407	39.6693
Whey protein	20.0000	19.9960	19.9601
α-Cornstarch	13.2000	13.1974	13.1737
Sucrose	10.0000	9.9980	9.9800
Soybean oil	7.0000	6.9986	6.9860
Cellulose	5.0000	4.9990	4.9900
Mineral mixture	3.5000	3.4993	3.4930
Vitamin mixture	1.0000	0.9998	0.9980
L-Cystine	0.3000	0.2999	0.2994
Choline bitartrate	0.2500	0.2500	0.2495
tert-Butylhidroquinone	0.0014	0.0014	0.0014
EMIQ	-	0.003	0.03
Total	100	100	100
Energy(Kcal/100 g)	373	373	372

### Immunohistochemical analysis

For immunohistochemical analysis, serial cross-sections (10-μm thick) of plantaris muscles were cut and stained with hematoxylin and eosin (H&E). The sections were observed using a fluorescent microscope (OLYMPUS BX51) and analyzed with the ImageJ software (the National Institutes of Health, USA) to calculate the mean cross-sectional area and the minimal fiber diameter. As described previously, cross-sectional areas of at least 100 randomly selected myofibers were measured [18]. Myofibers with central nuclei were excluded from analysis because they could be regenerated myofibers.

### Statistical analysis

All data are expressed as mean ± standard error of the mean (SEM). Statistical analysis was performed using the SPSS software (version 22). In Study 1, the groups were compared by two-way analysis of variance (ANOVA). One-way ANOVA was used in Study 2. Tukey’s test was performed for post-hoc comparisons. A *P*-value of <0.05 or <0.01 was considered to indicate a statistically significant difference.

## Results

### Study 1

The food intake, body weight, and plantaris muscle weight data are summarized in Table 3. The food intake and initial body weight were similar in all the groups. The final body weight was slightly lower in the Overload group compared to the Sham group (*P* < 0.05). In contrast, the weight of each plantaris muscle in the Overload groups was significantly higher than that in the Sham groups (*P* < 0.01). EMIQ supplementation slightly increased the plantaris muscle weight in the Overload + EMIQ and Sham + EMIQ groups, although the difference was not statistically significant (Fig. 1).

**Table 3:** Body and plantaris muscle weight in Study 1

	Sham	Sham + EMIQ	Ovld	Ovld + EMIQ		*P* value	
					Ovld	EMIQ	Ovld × EMIQ
Food intake (g day^−1^)	5.1 ± 0.2	5.2 ± 0.2	5.0 ± 0.2	5.0 ± 0.2	0.388	0.711	0.849
Initial body weight (g)	35.9 ± 0.5	35.5 ± 0.7	36.0 ± 0.5	35.0 ± 0.6	0.749	0.222	0.609
Final body weight (g)	39.3^*a*^ ± 0.6	39.7^*a*^ ± 0.7	38.1^*b*^ ± 0.9	37.6^*b*^ ± 0.6	0.032	0.964	0.547
Left Plantaris (mg)	21.6^*a*^ ± 1.0	22.7^*a*^ ± 0.9	43.4^*b*^ ± 2.3	46.0^*b*^ ± 3.2	0.000	0.388	0.727
Right Plantaris (mg)	21.8^*a*^ ± 0.9	23.3^*a*^ ± 0.8	41.5^*b*^ ± 3.0	45.3^*b*^ ± 3.7	0.000	0.298	0.638
Av. Plantaris (mg)	21.7^*a*^ ± 0.6	23.0^*a*^ ± 0.8	42.4^*b*^ ± 2.3	45.6^*b*^ ± 3.2	0.000	0.290	0.648
Plantaris/B.W. (%)	0.055^*a*^ ± 0.002	0.058^*a*^ ± 0.002	0.111^*b*^ ± 0.005	0.121^*b*^ ± 0.007	0.000	0.290	0.648

Values are mean ± SEM. Means without a common letter are statistically different (*P* < 0.05, two-way ANOVA (analysis of variance) followed by Tukey’s test). Sham: sham-operated mice, Sham + EMIQ: sham-operated mice receiving EMIQ, Ovld: functionally overloaded mice, Ovld + EMIQ: functionally overloaded mice receiving EMIQ

**Fig. 2:**
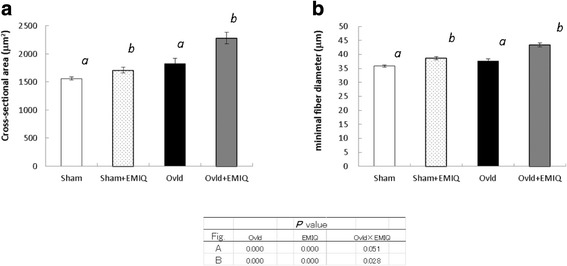
Cross-sectional area (**a**) and minimal fiber diameter (**b**) of the plantaris muscle in Study 1. Values are mean ± SEM. Means without a common letter are statistically different (*P* < 0.05, two-way ANOVA (analysis of variance) followed by Tukey’s test). Sham: sham-operated mice, Sham + EMIQ: sham-operated mice receiving EMIQ, Ovld: functionally overloaded mice, Ovld + EMIQ: functionally overloaded mice receiving EMIQ

To investigate the effect on the plantaris muscle, the cross-sectional area and minimal diameter of the myofibers were determined (Fig. 2). The cross-sectional area was 1559 ± 30 μm^2^, 1706 ± 51 μm^2^, 1820 ± 96 μm^2^, and 2278 ± 100 μm^2^ in the Sham, Sham + EMIQ, Overload, and Overload + EMIQ groups, respectively. The cross-sectional areas in the Overload groups were higher than those in the Sham groups (*P* < 0.01). The cross-sectional areas in the EMIQ-treated groups were higher than those in the untreated groups (*P* < 0.01). The minimal fiber diameter was 35.9 ± 0.4 μm, 38.6 ± 0.6 μm, 37.7 ± 0.7 μm, and 43.4 ± 0.7 μm in the Sham, Sham + EMIQ, Overload, and Overload + EMIQ groups, respectively. The minimal fiber diameters in the Overload groups were higher than those in the Sham groups (*P* < 0.01), and the minimal fiber diameters in the EMIQ-treated groups were higher than those in the untreated groups (*P* < 0.01). Two-way ANOVA detected an interaction between overload operation and EMIQ treatment.

### Study 2

To examine the cumulative effect of EMIQ/whey protein supplementation, we changed the protein source from milk casein to whey protein and examined overload-operated mice that received whey only and whey combined with EMIQ at two concentrations. The food intake, body weight, and plantaris muscle weight were similar in all the three groups (Table 4).

**Table 4:** Body and plantaris muscle weight in Study 2

	Ovld + W	Ovld + W + EMIQ(L)	Ovld + EMIQ(H)
Total food intake (g)	98.1 ± 2.3	99.0 ± 3.1	98.0 ± 1.6
Body mass (g)	37.2 ± 0.7	36.6 ± 0.8	37.3 ± 0.7
Change of body mass (g)	3.9 ± 0.7	2.9 ± 0.7	3.6 ± 0.4
Left Plantaris (mg)	46.9 ± 1.6	43.1 ± 2.6	43.0 ± 3.1
Right Plantaris (mg)	44.7 ± 4.3	46.3 ± 3.1	43.6 ± 1.7
Av. Plantaris (mg)	45.8 ± 2.9	44.7 ± 2.2	43.3 ± 2.1
Plantaris/B.W. (%)	0.124 ± 0.009	0.123 ± 0.008	0.117 ± 0.007

Values are mean ± SEM. Ovld + W: functionally overloaded mice receiving whey protein, Ovld + W + EMIQ (L): functionally overloaded mice receiving whey protein and EMIQ at a low concentration, Ovld + W + EMIQ (H): functionally overloaded mice receiving whey protein and EMIQ at a high concentration

**Fig. 3:**
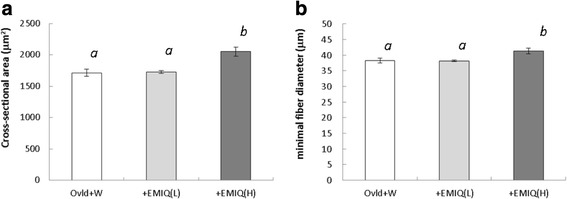
Cross-sectional area (**a**) and minimal fiber diameter (**b**) of the plantaris muscle in Study 2. Values are mean ± SEM. Means without a common letter are statistically different (*P* < 0.05, one-way ANOVA (analysis of variance) followed by Tukey’s test). Ovld + W: functionally overloaded mice receiving whey protein, +EMIQ (L): functionally overloaded mice receiving whey protein and EMIQ at a low concentration, +EMIQ (H): functionally overloaded mice receiving whey protein and EMIQ at a high concentration

To further investigate the effect on the plantaris muscle, the cross-sectional area and minimal diameter of myofibers were determined (Fig. 3). The cross-sectional area in the +EMIQ (H) group was significantly higher than in the Overload + W and +EMIQ (L) groups (*P* < 0.01; Overload + W: 1713 ± 58 μm^2^, +EMIQ (L): 1795 ± 114 μm^2^, +EMIQ (H): 2052 ± 73 μm^2^). Moreover, the minimal fiber diameter in the +EMIQ (H) group was higher than in the Overload + W and +EMIQ (L) groups (*P* < 0.05; Overload + W: 38.3 ± 0.8 μm, +EMIQ (L): 38.5 ± 1.4 μm, +EMIQ (H): 41.3 ± 0.9 μm).

## Discussion

The present study demonstrated that EMIQ supplementation intensifies muscle fiber hypertrophy in functionally overloaded mice. Moreover, EMIQ supplementation in sham-operated mice and EMIQ/whey protein supplementation also intensified muscle fiber hypertrophy in this model. Quercetin has been reported to prevent disused muscle atrophy [3, 5]. EMIQ is a quercetin with improved absorption. In the present study, we showed that EMIQ can stimulate muscle fiber hypertrophy. Few studies have found that food ingredients, except proteins, amino acids, and their metabolites, can induce muscle hypertrophy [19–21].

One possible mechanism of muscle fiber hypertrophy stimulation by EMIQ is via its antioxidant activity. Functional overlord and eccentric exercise induce inflammation and oxidative stress in the muscle. According to many reports, antioxidant supplementation can reduce the inflammation and oxidative damage, although it is unclear whether supplementation of antioxidants influences muscle hypertrophy [22]. Antioxidants are expected to prevent muscle damage, oxidative stress, and muscular fatigue, leading to prolonged exercising capacity and muscle hypertrophy. On the contrary, reactive oxygen species activates important cell signaling pathways that mediate skeletal muscle adaptions to exercise, such as hypertrophy [23]. Vitamin C, a common antioxidant, has been reported to attenuate overload-induced skeletal muscle hypertrophy [24], though an antioxidant mixture including rutin promoted muscle protein synthesis by restoring the impaired leucine stimulation [25]. Therefore, specific antioxidants may positively affect muscle hypertrophy.

Another possible mechanism is related to the effects of rutin on mitochondria. Recent studies have suggested that quercetin exerts its beneficial effects independently of its antioxidant activity. Thus, quercetin modulates pathways related to mitochondria biogenesis, elevation of mitochondria membrane potential, oxidative respiration and ATP anabolism, and intra-mitochondrial redox status [26]. Muscle protein synthesis is a process that requires high utilization of ATP and mitochondria is an important regulator of intracellular signaling cascades that modulate skeletal muscle size and function [27]. Dysfunctional mitochondria trigger proteolytic pathways leading to muscle atrophy during aging. In contrast, exercise training can induce mitochondrial biogenesis and dynamics, and increase muscle protein synthesis that favors myofiber and whole muscle hypertrophy. Overexpression of ATP citrate lyase, which improves mitochondrial function, is sufficient to induce hypertrophy in human myotubes [28]. It is therefore possible that EMIQ induces muscle fiber hypertrophy by affecting mitochondria, although the detailed mechanism needs to be explored in the future.

A remarkable finding was that EMIQ supplementation induced muscle fiber hypertrophy even in the sham-operated group. This result indicates that EMIQ intake may induce muscle fiber hypertrophy without functional overload mimicking resistant training. Moreover, EMIQ supplementation combined with whey protein supplementation also induced muscle fiber hypertrophy in overload-operated mice. Protein intake after resistance exercise augments muscle protein synthesis and can lead to muscle fiber hypertrophy. Whey protein, which contains high amounts of essential branched amino acids and is absorbed rapidly, is especially effective in this regard [12, 13], resulting in its widespread use by athletes. Our results suggest that EMIQ supplementation might have a beneficial effect in athletes; however, it needs to be validated in a human study.

It has to be noted that the effective concentration of EMIQ in Study 2 was 10-fold higher than in Study 1, suggesting that effective EMIQ concentration depends on the food content. Whey contained in Study 2 is reported to augment muscle hypertrophy dependent on high content of leucine. Therefore, it is speculated that the amount of EMIQ to be effective needs to be high. Study 1 identified an interaction between overload operation and EMIQ treatment, indicating that the effective concentration of EMIQ is affected by presence or absence of functional overload. We speculate that the effective concentration of EMIQ may depend on specific details of exercise and/or nutrition. EMIQ can induce muscle fiber hypertrophy in resistance training and daily life. Additional studies will be needed to determine the exact mechanisms and conditions.

## Conclusions

EMIQ supplementation intensified the fiber hypertrophy of the plantaris muscle in mice induced by compensatory overload. Moreover, both EMIQ supplementation alone and in combination with whey protein intake increased muscle fiber hypertrophy. Given that muscle fiber hypertrophy is beneficial not only in athletes but also in the elderly with locomotive syndrome, EMIQ can be an effective supplement for various sub-populations in need of muscle fiber hypertrophy and maintenance.

## Abbreviations

+EMIQ (H): overload-operated mice that received whey protein and a high dose of EMIQ
+EMIQ (L): overload-operated mice that received whey protein and a low dose of EMIQ
ANOVA: analysis of variance
EMIQ: enzymatically modified isoquercitrin
Overload + EMIQ: overload-operated mice that received EMIQ
Overload + W: overload-operated control mice that received whey protein
Overload: overload-operated control mice
Sham + EMIQ: sham-operated mice that received EMIQ
Sham: sham-operated control mice
